# Antioxidant and Anti-Inflammatory Properties of *Buddleja globosa* Hope (Matico): A Systematic Review of Phytochemical Composition, Molecular Mechanisms, and Translational Evidence

**DOI:** 10.3390/antiox15070790

**Published:** 2026-06-24

**Authors:** Álvaro Becerra, Felipe Soto, Daniela Millán, Juan José Valenzuela-Fuenzalida, Maria P. Moya, José E. León-Rojas, Manuel E. Cortés

**Affiliations:** 1 Escuela de Fonoaudiología & Departamento de Ciencias Químicas y Biológicas, Facultad de Ciencias de la Salud, Universidad Bernardo O’Higgins, Santiago 8370854, Chile; alvaro.becerra@ubo.cl (Á.B.); f.soto.guerrero@gmail.com (F.S.); 2 Escuela de Nutrición y Dietética & Centro Integrativo de Biología y Química Aplicada (CIBQA), Facultad de Ciencias de la Salud, Universidad Bernardo O’Higgins, Santiago 8370854, Chile; daniela.millan@ubo.cl; 3 Departamento de Morfología, Facultad de Medicina, Universidad Andrés Bello, Santiago 8370146, Chile; 4 Facultad de Ciencias de la Salud, Universidad Autónoma de Chile, Santiago 7500912, Chile; maria.moya@uautonoma.cl; 5 Grupo de Investigación Bienestar, Salud y Sociedad, Escuela de Psicología y Educación, Universidad de Las Américas, Quito 170124, Ecuador; 6 Dirección de Investigación, Vicerrectoría Académica, Universidad Bernardo O’Higgins, Santiago 8370854, Chile; manuel.cortes@ubo.cl

**Keywords:** anti-inflammatory, antioxidant, *Buddleja globosa*, matico, phenylethanoid glycosides, verbascoside, systematic review, translational pharmacology

## Abstract

**Background:** *Buddleja globosa* Hope (matico) is a Chilean medicinal plant traditionally used in Mapuche and Aymara ethnomedicine. However, no systematic synthesis of its phytochemical composition and pharmacological evidence has been previously reported. **Methods:** A PRISMA 2020-compliant systematic review was conducted using Google Scholar, PubMed, EBSCOhost, and Springer Nature databases from inception to March 2026. Studies reporting phytochemical characterization and/or biological activities of ***B. globosa*** were included. Methodological quality was assessed using an adapted five-criterion tool for non-clinical studies. The protocol was registered in OSF. **Results:** Fourteen studies (1989–2026), mainly from Chilean research groups, identified 27 bioactive compounds across leaves, roots, and flowers. These included phenylethanoid glycosides (e.g., verbascoside/acteoside, echinacoside, forsitoside B, and linarin), flavonoids (luteolin 7-O-glucoside, apigenin 7-O-glucoside, myricetin, catechin, and epicatechin), pentacyclic triterpenes (α/β-amyrins and β-sitosterol), iridoid glycosides, and clerodane diterpenoids (buddledines A–C), as well as four newly reported phenylethanoids. Antioxidant activity was the most frequently evaluated endpoint (11/14 studies), mainly mediated through hydrogen atom transfer and single-electron transfer mechanisms linked to caffeoyl and flavonoid structures. Anti-inflammatory effects (five studies) involved COX and 5-LOX inhibition and reduced PGE_2_ production in LPS-stimulated macrophages. Additional reported activities included antihepatotoxic, antiplatelet, wound-healing, antibacterial, and antifungal effects. **Conclusions:** ***B. globosa*** exhibits a coherent phytochemical profile supporting strong preclinical antioxidant and anti-inflammatory activities. The main limitation for clinical translation is the low oral bioavailability of phenylethanoid glycosides. Nanoformulation strategies, investigation of colonic metabolites, and topical delivery systems represent promising approaches to bridge the preclinical-to-clinical gap.

## 1. Introduction

Ethnopharmacology has gained considerable scientific traction over the past two decades, driven by the recognition that medicinal plants represent a largely underexplored reservoir of structurally diverse bioactive compounds [1,2]. The natural flora of the Southern Cone encompasses approximately 12,000 species distributed across eco-regions, including the Andes Cordillera, the Valdivian and Subantarctic temperate forests, and the Patagonian steppe, each shaped by distinct environmental pressures [1]. Medicinal and aromatic plants constitute an important source not only of herbal medicines but also of plant-derived materials with applications across multiple industrial sectors, and their systematic exploration has expanded steadily in recent years [1,2].

**Figure 1:**
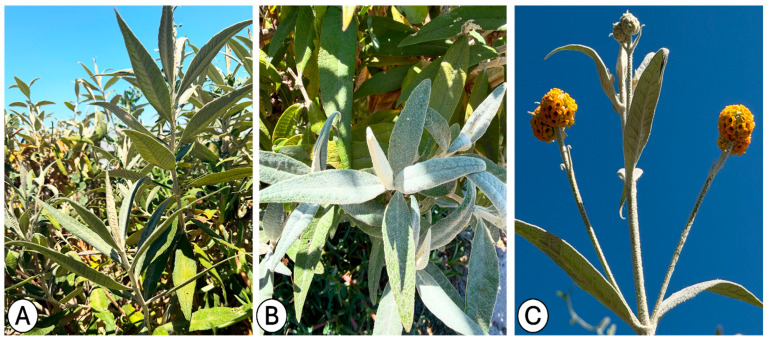
Matico (***Buddleja globosa*** Hope) is widely used in traditional Chilean medicine: (**A**) ***B. globosa*** is an evergreen dioecious shrub with yellowish, tomentose, and robust stems, reaching up to 4 m in height. This species is common in central and southern Chile [15]. (**B**) Branches are tomentose, and the opposite leaves are subsessile, lanceolate to elliptic, subcoriaceous to membranaceous, glabrescent, and decurrent to the base; blades measure 3–15 cm in length by 1–5 cm in width, with a rough adaxial surface and a whitish tomentose abaxial surface, and an acute apex [3]. (**C**) Inflorescences are globose heads (1–2 cm in diameter) composed of tubular flowers varying from yellow to orange and red [3]. Source: Images taken by the authors during summer in Alto Macul, La Florida, Santiago of Chile, at approximately 900 m a.s.l.

***Buddleja globosa*** **(*****B. globosa*****)** Hope stands among the most pharmacologically relevant medicinal plants of this floristic context [3]. The genus *Buddleja* comprises approximately 100 species of trees and shrubs distributed across tropical and subtropical regions of the Americas, Africa, and Asia, several of which are integrated into traditional healing systems [3,4]. In Chile, ***B. globosa***, popularly known as *matico,* also *pañil* or *palnguiñ* in the Mapundungun language, referring to a shrub growing in a sunny place, is the only native species of the genus with systematic traditional medicinal use, included in the first edition of the Chilean Pharmacopoeia in 1886 [3,5]. Botanically, it is a dioecious shrub reaching 1.5–4 m in height (Figure 1A), with tomentose branches, subsessile opposite leaves that are oval-lanceolate, rough on the adaxial surface and whitish beneath (Figure 1B), and globose inflorescences (Figure 1C) composed of yellow-orange tubular flowers [3]. Its leaves are the principal therapeutic organ used in folk medicine, both as topical poultices for wounds and ulcers and as aqueous infusions for gastrointestinal and hepatobiliary complaints [3,4,5]. The ethnobotanical relevance of ***B. globosa*** extends across multiple indigenous (e.g., Aymara, Diaguita and Mapuche) and rural traditions throughout Chile and across both Chilean and Argentine Patagonia [6,7,8]. In northern Chile, Diaguita ethnobotanical practice incorporates the species into its local therapeutic repertoire [6,8]. Likewise, among Mapuche communities of north-western Patagonia, the species is systematically collected for wound treatment and as an anti-inflammatory within a broader framework of transmitted plant knowledge [7]. Beyond the ancestral indigenous use, matico ranks among the most frequently self-prescribed medicinal plants by older adults attending primary healthcare centers in Chile [9].

Experimental pharmacology has progressively validated these traditional uses [10,11,12]. Preclinical studies have demonstrated anti-inflammatory effects through dual inhibition of cyclooxygenase (COX) and 5-lipoxygenase (5-LOX) pathways [13,14], antinociceptive activity across chemical-inflammatory and visceral pain models [15], and significant protection of human dermal fibroblasts against hydrogen peroxide-induced oxidative injury at concentrations as low as 2.5 µg/mL [16]. The principal bioactive constituents include the phenylethanoid glycosides verbascoside (acteoside) and echinacoside, the flavone derivatives luteolin 7-O-glucoside and linarin, and terpenoids such as buddledine A, with verbascoside consistently emerging as the dominant phenolic marker in leaf material [10,12,17,18]. High-resolution quadrupole time-of-flight mass spectrometry QTOF/MS analyses have expanded the known metabolite profile, identifying β-hydroxy-verbascoside, campneoside I, martynoside, and additional flavone glycosides [11].

Despite this accumulated evidence, the molecular mechanisms underlying the biological effects of ***B. globosa*** phenolic compounds remain incompletely characterized [10,11,12,17,18]. In particular, the capacity of its phenylethanoids and flavonoids to modulate redox-dependent cell signaling pathways governed by reactive oxygen and nitrogen species (ROS/RNS) has not been systematically examined [19,20]. This gap is mechanistically significant, given that catechol and ortho-diphenol polyphenols, including verbascoside and luteolin, are recognized modulators of the Nrf2/Keap1 axis, NF-κB suppression, and neuroprotective gene expression [19,21].

The guiding research question of this systematic review was: What phytochemical constituents and experimentally validated biological activities have been reported for ***B. globosa*****,** and which molecular mechanisms may explain its antioxidant and anti-inflammatory properties? This review was guided by the hypothesis that the ethnomedicinal uses of ***B. globosa*** are pharmacologically supported by a coherent phytochemical profile characterized by phenylethanoid glycosides and flavonoids that modulate oxidative and inflammatory pathways.

In view of the aforementioned evidence, this systematic review aims to compile and critically evaluate the phytochemical and pharmacological evidence for ***B. globosa***, and to analyze the capacity of its constituents to modulate ROS/RNS-dependent pathways associated with anti-inflammatory and antioxidant mechanisms, with the objective of identifying priority research directions and translational opportunities for this ethnopharmacologically relevant Chilean medicinal plant [1,3,20,21].

## 2. Materials and Methods

### 2.1. Study Design

This study was conducted within a framework for synthesizing evidence that integrated phytochemical, pharmacological and ethnobotanical data. This review was conducted in accordance with the Preferred Reporting Items for Systematic Reviews and Meta-Analyses (PRISMA) 2020 checklist [22]. The review protocol was prospectively registered in the Open Science Framework (OSF): https://osf.io/9hcr7 (accessed on 18 April 2026). No ethical approval was required, as the study involves neither human participants nor animal experiments.

### 2.2. Eligibility Criteria

Studies were considered eligible if they met the following criteria: (i) original research articles published in peer-reviewed journals; (ii) studies specifically focused on ***B. globosa*** Hope; (iii) reports containing phytochemical characterization and/or antioxidant, anti-inflammatory, wound-healing, antimicrobial, antiplatelet, hepatoprotective, or related pharmacological activities; and (iv) articles published in English. No restriction on publication year was applied.

Exclusion criteria comprised: (i) review articles, book chapters, conference abstracts, editorials, and theses; (ii) studies not specifically involving ***B. globosa*****;** (iii) articles lacking phytochemical or biological activity data relevant to the objectives of this review; and (iv) studies for which the full text could not be retrieved.

The review question was formulated using a framework inspired by the PECO model, adapted for non-clinical experimental research. Within this framework, ***B. globosa*** extracts and isolated phytochemicals were considered the experimental interventions, compared against appropriate controls or reference compounds, with outcomes focused on antioxidant, anti-inflammatory, and related pharmacological activities.

Although only original studies published in English were eligible for systematic inclusion, complementary background and discussion contextualization also incorporated relevant scientific literature published in Spanish.

### 2.3. Information Sources and Search Strategy

Database searches were primarily conducted in PubMed, EBSCOhost, and Springer Nature, with Google Scholar used as a complementary source to maximize retrieval of ethnopharmacological and phytochemical literature potentially underrepresented in indexed biomedical databases during March 2026, covering all publications available to date. The Boolean search string employed was: (“phytochemistry OR “phenolic compounds” OR flavonoids OR antioxidant OR anti-inflammatory OR pharmacology OR bioactivity OR wound healing OR acteoside OR verbascoside “ AND “matico” OR “*Buddleja globosa*”). Additional records were identified through manual screening of reference lists of included studies.

### 2.4. Study Selection

All records were imported into a reference manager and duplicates removed. Two independent reviewers screened titles and abstracts, followed by full-text assessment of potentially eligible articles. Disagreements at any stage were resolved by consensus. No automation tools were employed during the selection process.

### 2.5. Data Extraction

Data extracted from each included study comprised the following: publication year, country of origin, plant part analyzed, extraction methodology, analytical platform employed for phytochemical characterization, identified compounds, experimental model, biological assays, principal pharmacological outcomes, and proposed molecular mechanisms of action.

### 2.6. Structural Visualization

Two-dimensional chemical structures of selected bioactive compounds were retrieved from the PubChem open chemistry database (National Center for Biotechnology Information, U.S. National Library of Medicine; https://pubchem.ncbi.nlm.nih.gov; accessed on 20 April 2026) and incorporated into figures to provide structural reference for the bioactive compounds identified across the included studies. No structural modifications were made to the original depictions.

## 3. Results

### 3.1. Study Selection

**Figure 2:**
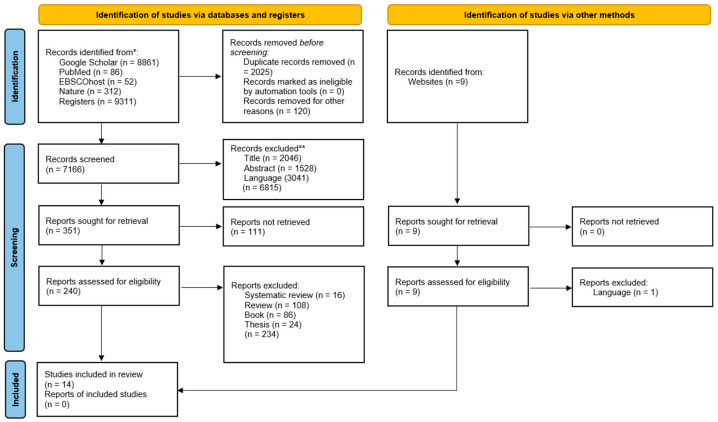
PRISMA 2020 flow diagram illustrating the selection of studies included in the review. Adapted from Page et al. (2021) [22]. A double asterisk indicates complete consensus among the authors; a single asterisk would indicate partial agreement on the search, which should be reviewed by an additional reviewer.

A total of 9311 records were identified through database searches: Google Scholar (*n* = 8861), Springer Nature (*n* = 312), PubMed (*n* = 86), and EBSCOhost (*n* = 52). Prior to formal screening, 2025 duplicate records were removed, and 120 records were excluded for other reasons, leaving 7166 unique records for title and abstract screening. Of these, 6815 records were excluded for the following reasons: irrelevant title or scope (*n* = 2046), abstracts without relevant pharmacological or phytochemical data (*n* = 1728), and language other than English (*n* = 3041), leaving 351 articles assessed for eligibility. Of these, 111 could not be retrieved, yielding 240 articles for full-text review. After applying predefined inclusion and exclusion criteria, 234 articles were excluded: narrative reviews (*n* = 108), book chapters (*n* = 86), systematic reviews (*n* = 16), and theses (*n* = 24). In addition, 9 records were identified through manual screening of reference lists of included studies, of which 1 was excluded due to language other than English, yielding 8 additional eligible studies. Thus, a total of 14 original articles were included in this systematic review. The PRISMA 2020 flow diagram is presented in Figure 2. All 14 included studies are original research articles written in English, consisting exclusively of in vitro and/or in vivo laboratory work, with no clinical interventions on humans. Studies originated primarily from Chile (n = 9), the United Kingdom (n = 3), and Portugal (n = 2). The main characteristics of the included studies are summarized in Table 1. Twelve of 14 studies provided voucher specimen information or clear botanical collection data; all studies employed at least one validated analytical technique; and 11 of 14 reported appropriate positive and negative controls in bioassays. Statistical power was formally declared in four studies. The two studies with the lowest methodological scores were Houghton et al. [23] and Suwalsky et al. [24], due to limited quantification of individual compounds and the absence of explicit power calculations, respectively. Overall, the methodological quality of the included evidence was considered acceptable for phytochemical systematic reviews, consistent with standards applied in comparable publications in this journal. The complete absence of clinical studies precludes any assessment of clinical risk of bias.

### 3.2. Phytochemical Characterization and Extraction Conditions

Phytochemical analyses across the 14 included studies revealed a structurally diverse range of 27 secondary metabolites spanning eight chemical classes: phenylethanoid glycosides, flavones, flavonols, flavan-3-ols, flavanones, iridoid glycosides, terpenoids (sesquiterpenoids, diterpenoids, and triterpenoids), and phytosterols. The most frequently identified compounds were verbascoside (acteoside), echinacoside, forsitoside B, luteolin, and luteolin 7-*O*-glucoside, detected across multiple independent studies using different extraction procedures and analytical platforms (Table 1 and Table 2).

Extraction methods varied considerably across the study period. The earliest studies employed aqueous extractions (boiling water or aqueous infusion) [16,18,23], yielding fractions enriched in polar compounds. Studies aimed at full chemical characterization adopted sequential extraction with solvents of increasing polarity (*n*-hexane, dichloromethane, methanol) [11,14,15]. Methanolic and hydroalcoholic extractions (EtOH:H_2_O 50:50–70:30 *v*/*v*) predominated in more recent research [10,12,17,24,25,26,27]. Ultrasound-assisted extraction was incorporated by Hernández et al. [12] and Hermosilla et al. [27]; the extraction yield in the latter was approximately 20% across four seasonal collection variants (M1–M4).

Analytical identification evolved from TLC, UV/IR spectrophotometry, and ^1^H NMR in early studies [13,16,18] to HPLC-DAD [11,14,15,25], LC-ESI-MS/MS [11,15,25], UHPLC-DAD-ESI/MS [10,12,26], and high-resolution HPLC-ESI/MS platforms with mass accuracy below 3.5 ppm [27]. In parallel, Suwalsky et al. [24] integrated biophysical membrane approaches, including small-angle and wide-angle X-ray scattering (SAXS/WAXS and differential scanning calorimetry (DSC), to evaluate interactions between *B. globosa* extracts and phospholipid bilayer systems.

### 3.3. Identified Compounds and Quantitative Data

A total of 27 distinct bioactive compounds or compound groups were identified (Table 2). Among phenylethanoid glycosides, verbascoside (acteoside) was the most consistently quantified compound, with concentrations ranging from 63.1 mg/g dry weight (DW) (aqueous infusion; Gastaldi et al. [8]) to 112.4 ± 5.1 mg/g DW (methanolic fractionation by centrifugal partition chromatography; Torres et al. [11]) and 98.7 mg/g DW (ultrasound-assisted 70% EtOH; Hernández et al. [12]). Echinacoside was quantified at 75 ± 3.2 mg/g DW [10] and previously isolated in 125 mg yield from 50 g fresh leaves [18]. Forsitoside B was quantified at 55.4 mg/g DW [15] and detected as a major HPLC peak in subsequent studies [11,12,27]. Seasonal variation was documented for verbascoside [15], with autumn collections yielding approximately half the concentration observed in summer; a finding with direct implications for botanical standardization.

Among flavones and flavonols, linarin was quantified at 58 ± 2.1 mg/g DW [10], while luteolin 7-O-glucoside and apigenin 7-O-glucoside were consistently detected as major constituents of the methanolic leaf fraction [11,12,14,15]. In flower extracts [26], catechin was the predominant compound (682.4 ± 21.3 mg/100 g DW), with quercitrin (58.3 mg/100 g DW) and pinocembrin exhibiting a Variable Importance in Projection value > 1 in Partial Least Squares regression analysis. Total polyphenol content ranged from 147.2 ± 4.5 mg GAE/g DW [8] to 183.4 ± 7.2 mg GAE/g DW [12].

Houghton and Hikino [18] isolated eight iridoid glycosides, including two novel iridoids (6-p-methoxycinnamoylaucubin and 6-p-methoxycinnamoylcatalpol) not previously reported in the genus *Buddleja*. From the lipophilic fractions, α- and β-amyrins and β-sitosterol were isolated from hexane and dichloromethane extracts [14]. Liao et al. [13] isolated novel clerodane diterpenoids (buddledines A, B, C; dihydrobuddledine A) and the sesquiterpene zerumbone from chloroform root extracts, representing the first report of these compound classes in *B. globosa.* Hermosilla et al. [27] identified four phenylethanoid glycosides not previously reported for the species: Betonioside E (C_35_H_46_O_20_; [M−H]^−^ *m*/*z* 785.3; error 1.38 ppm), β-OH-forsitoside B methyl ether (*m*/*z* 785.3; error 1.35 ppm), samioside (C_34_H_44_O_19_; *m*/*z* 755.2), and stachyoside A (C_34_H_44_O_19_; *m*/*z* 755.2), all confirmed by HPLC-ESI/MS with mass errors below 3.5 ppm.

### 3.4. Biological Activities and Assay Conditions

The 14 included studies reported a broad range of biological activities in *B. globosa* extracts and isolated compounds, including antioxidant, anti-inflammatory, analgesic, antihepatotoxic, wound-healing, antiplatelet, antimicrobial, and antifungal effects (Table 1 and Table 2).

#### 3.4.1. Antioxidant Activity

Antioxidant activity was the most widely evaluated endpoint, assessed in 11 of 14 studies. The 2,2-diphenyl-1-picrylhydrazyl (DPPH) radical scavenging served as the primary assay, complemented by 2,2′-azinobis-(3-ethylbenzothiazoline-6-sulfonic acid) (ABTS) [12], oxygen radical absorbance capacity (ORAC) [25,26], thiobarbituric acid reactive substances/ malondialdehyde (TBARS/MDA) [14,16], and xanthine oxidase inhibition [14]. The total methanolic extract exhibited a DPPH radical scavenging IC_50_ of 8.4 µg/mL [14], while the sequential methanolic extract demonstrated a lower IC_50_ value of 6.0 µg/mL. In comparison, quercetin, used as the reference antioxidant standard, exhibited an IC_50_ of 1.07 µg/mL. Gastaldi et al. [25] reported DPPH and ORAC activity attributable primarily to verbascoside and forsitoside B. Hernández et al. [12] obtained the highest total polyphenol content in the review, with DPPH and ABTS activity correlated with verbascoside content.

In flower extracts, Zamorano-Aguilar et al. [26] identified catechin, myricetin, and pinocembrin as the primary antioxidant contributors. Significant reduction in intracellular ROS was confirmed in CHO-K1 cells at 5–50 µg/mL. Suwalsky et al. [24] uniquely characterized antioxidant effects at the membrane level using SAXS/WAXS: *B. globosa* extracts protected DPPC/DMPC phospholipid bilayers against oxidative structural disruption, and H_2_O_2_-induced haemolysis in human erythrocytes was significantly attenuated, providing biophysical evidence of lipid bilayer-protective antioxidant activity with direct relevance to chronic disease pathophysiology.

#### 3.4.2. Anti-Inflammatory Activity

Anti-inflammatory activity was evaluated in five studies [11,12,13,14,15] employing in vitro enzyme inhibition assays and in vivo animal models. Liao et al. [13] reported the highest anti-inflammatory potency among the included studies: buddledines and zerumbone produced 100% inhibition of 5-LOX and 86.5 ± 5.5% inhibition of COX at 50 µg/mL, as measured by [^14^C]-arachidonic acid metabolism in rat peritoneal leukocytes. This dual COX/5-LOX inhibition is pharmacologically significant because simultaneous blockade of both enzymes prevents compensatory arachidonic acid shunting toward alternative inflammatory mediators, a recognized limitation of selective COX-2 inhibitors [20,21].

Backhouse et al. [14] validated anti-inflammatory activity through bioassay-guided fractionation in vivo: the total methanolic extract produced 61.4% inhibition in the carrageenan-induced paw edema model in guinea pigs. The α/β-amyrin fraction inhibited AA-induced ear edema by 47.7% and TPA-induced ear edema by 79.0%. The phytosterol fractions CD4-N and CD5-N exhibited TPA-induced anti-inflammatory inhibition of 78.2% and 83.7%, respectively, at 1 mg/20 µL per ear, exceeding that of nimesulide at an equivalent dose. Backhouse et al. [15] subsequently characterized the antinociceptive and COX-inhibitory activity of the verbascoside-enriched fraction; verbascoside demonstrated superior antinociceptive potency relative to ibuprofen in the writhing test, with pharmacological antagonism by naloxone, cyproheptadine, and prazosin indicating convergent opioidergic, serotonergic, and adrenergic pathway participation.

Torres et al. [11] demonstrated PGE_2_ inhibition in LPS-stimulated RAW 264.7 macrophages attributable to the phenylethanoid glycoside fraction, principally verbascoside, forsitoside B, and luteolin 7-O-glucoside. This finding provides direct cellular-level evidence of suppression of inflammatory mediators, substantially reinforcing the translational relevance of the anti-inflammatory data obtained from in vivo models.

#### 3.4.3. Additional Biological Activities

Analgesic activity was demonstrated in two studies [14,15]. Backhouse et al. [14] reported analgesic effects of 41.2% for α/β-amyrins and 38.5% for the methanolic extract. Antihepatotoxic activity was uniquely characterized by Houghton and Hikino [18] in cultured rat hepatocytes: echinacoside produced ~45% GPT reduction at 1.0 µg/mL against galactosamine (GalN) and complement-mediated cytotoxic system (CMC); linarin showed ~41% GPT reduction versus GalN and ~74% versus CMC; the first report of antihepatotoxic activity for this compound. The novel iridoid 6-p-methoxycinnamoylaucubin produced ~44% GPT reduction versus GalN (*p* < 0.001), while aucubin was inactive in the cultured hepatocyte system, suggesting that aucubin’s in vivo activity requires metabolic biotransformation.

Wound-healing activities were documented by Mensah et al. [16] (H_2_O_2_-protective effects; MDA reduction in dermal fibroblasts) and Houghton et al. [23] (keratinocyte differentiation induction). Antiplatelet activity was characterized by Fuentes et al. [10] via PLC-γ2 and PKC-β2 inhibition and calcium mobilization disruption in human platelet-rich plasma. Antifungal activity against *C. albicans* (log reduction 4.1 in PCL nanofiber formulation P4; cytocompatibility ~130% viability in 3T3-L1 fibroblasts; no antibacterial activity vs. *S. aureus* or *P. aeruginosa* observed in this study) was reported exclusively by Hermosilla et al. [27]. Antibacterial activity against *S. aureus*, *E. coli*, and *P. aeruginosa* (broth microdilution MIC) was reported by Hernández et al. [12]. Antibacterial activity against *S. aureus* and *P. aeruginosa*, assessed via a chitosan/hyaluronic acid/gelatin scaffold platform optimized by Box–Behnken design, was reported by Ceriani et al. [17].

### 3.5. Molecular Mechanisms of Action

The molecular mechanisms underlying the biological activities of *B. globosa* bioactive compounds are summarized in Table 2.

#### 3.5.1. Antioxidant Mechanisms

In phenylethanoid glycosides, antioxidant activity is primarily attributed to the ortho-dihydroxyl (catechol) moiety of the caffeoyl residue, enabling HAT and SET mechanisms. Verbascoside additionally protects erythrocyte membranes by reducing oxidative stress-mediated lipid peroxidation [24], demonstrated biophysically by SAXS/WAXS and haemolysis assays. The glucosyl units modulate aqueous solubility and pharmacokinetic behavior but do not directly contribute to radical scavenging [28,29].

For flavones and flavonols, antioxidant activity depends on the catechol B-ring configuration (luteolin, quercetin), while metal chelation (Fe^2+^, Cu^2+^) via the 3-OH/4-keto chelating site and xanthine oxidase inhibition contribute additional mechanisms [14]. The 3′,4′,5′-trihydroxy B-ring of myricetin enables multiple sequential HAT events per molecule, accounting for its superior radical-scavenging potency [26,30]. Catechin and epicatechin additionally chelate Fe^2+^ to prevent Fenton-type hydroxyl radical generation and exhibit cytoprotective activity in CHO-K1 cells [26].

At the membrane level, *B. globosa* extracts intercalate into the polar head region of DPPC/DMPC phospholipid bilayers, modifying lamellar periodicity in a concentration-dependent manner [24]. This membrane-protective mechanism is directly relevant to in vivo lipid peroxidation in chronic inflammatory and metabolic diseases.

#### 3.5.2. Anti-Inflammatory Mechanisms

Anti-inflammatory mechanisms in *B. globosa* involve multiple convergent molecular pathways. Within the arachidonic acid cascade, buddledines and zerumbone produce dual COX/5-LOX inhibition [13]; α/β-amyrins act through phospholipase A_2_ inhibition, with NF-κB/MAPK pathway suppression proposed as the upstream mechanism based on evidence from related triterpene systems; this pathway was not directly measured in *B. globosa* [14,20]; and the phytosterol fractions (CD4-N/CD5-N, containing β-sitosterol and β-sitosterol glucoside) produce 78.2–83.7% topical anti-inflammatory inhibition in the TPA-ear edema model, exceeding nimesulide at equivalent dose [14].

Among polar phenolic compounds, luteolin and luteolin 7-O-glucoside inhibit inducible COX-2 and iNOS, reducing PGE_2_ and NO production in inflamed tissue [11,15]. Luteolin 7-O-glucoside has been shown to inhibit MMP-2 and MMP-9 in independent experimental systems, suggesting a potential role in attenuating extracellular matrix degradation in inflammatory lesions [11,15]. Verbascoside inhibits iNOS-dependent NO production in LPS-stimulated cells and suppresses histamine- and bradykinin-induced smooth muscle contractions in vitro [15]. Torres et al. [11] directly confirmed PGE_2_ suppression in LPS-stimulated RAW 264.7 macrophages for the phenylethanoid glycoside fraction, consistent with the NF-κB inhibitory activity of acteoside documented in independent experimental systems [31,32]; however, the specific intracellular pathway was not directly measured in that study, and mechanistic attribution to NF-κB must therefore be qualified as biologically plausible but not directly confirmed within the included literature.

Antinociceptive mechanisms involve partial modulation of µ, δ, and κ opioid receptors, 5-HT3 serotonergic receptors, and α-1 adrenoceptors, as documented by pharmacological antagonism studies employing naloxone, cyproheptadine, and prazosin in Backhouse et al. [15]. The convergence of opioidergic, serotonergic, and adrenergic pathway participation in verbascoside’s antinociceptive effect (surpassing ibuprofen at equimolar doses) warrants further receptor-level characterization.

#### 3.5.3. Mechanisms of Additional Biological Activities

Antihepatotoxic mechanisms of echinacoside and linarin are attributed to catechol moiety-driven reduction in oxidative stress in hepatocytes and stabilization of hepatocyte membrane integrity against complement- and toxin-mediated cytotoxicity [18]. The iridoid 6-p-methoxycinnamoylaucubin likely acts through a distinct mechanism, given that aucubin requires in vivo biotransformation for hepatoprotective activity [18]. Antiplatelet mechanisms of verbascoside involve inhibition of PLC-γ2/PKC-β2 signaling and disruption of calcium mobilization in collagen- and TxA_2_-activated platelets [6]. For the four newly identified phenylethanoid glycosides in Hermosilla et al. [27] (betonioside E, β-OH-forsitoside B methyl ether, samioside, stachyoside A), mechanistic data in *B. globosa* are not yet available; proposed activities are inferred from structural analogy with related phenylethanoids and must be regarded as working hypotheses pending direct pharmacological characterization.

## 4. Discussion

This systematic review consolidates, for the first time, the complete pharmacological and phytochemical evidence base for *B. globosa* through 14 primary studies that identified 27 compounds in the leaves, roots, and flowers, spanning almost four decades of predominantly Chilean research. The reviewed evidence confirms a structurally diverse and pharmacologically coherent phytochemical profile, dominated by phenylethanoid glycosides, flavonoid glycosides, and terpenoids, whose biological activities mainly converge on antioxidant and anti-inflammatory endpoints [20,21,33,34].

### 4.1. Antioxidant Activity: Mechanistic Convergence and Structural Basis

Antioxidant activity was evaluated in 11 of 14 studies, constituting the most consistently assessed pharmacological endpoint in this evidence base. Multi-platform corroboration: DPPH, ABTS, ORAC, TBARS/MDA, and H_2_O_2_-induced haemolysis consistently attribute the antioxidant capacity of polar *B. globosa* fractions to the ortho-dihydroxyl (catechol) moiety of the caffeoyl residue in verbascoside and echinacoside, thereby enabling HAT and SET mechanisms [28,34]. The glucosyl units modulate aqueous solubility and pharmacokinetic behavior but do not directly contribute to radical scavenging [28,29].

Suwalsky et al. [24] extended this characterization to the membrane level, demonstrating through SAXS/WAXS that *B. globosa* extracts protect DPPC/DMPC phospholipid bilayers against H_2_O_2_-induced structural disruption and significantly attenuate erythrocyte haemolysis; a mechanism directly relevant to lipid peroxidation in chronic diseases [35]. This biophysical dimension of antioxidant activity, absent from purely chemical assay-based studies, provides a higher-fidelity model of physiological antioxidant protection not employed in the recent review by Becerra et al. [33] about *P. quinquefolia* (Virginia creeper), reinforcing the added mechanistic depth provided by the *B. globosa* evidence base.

In flower extracts, myricetin and catechin are the primary antioxidant contributors, with cytoprotective activity confirmed in CHO-K1 cells [26]. The trihydroxylated B-ring of myricetin enables multiple sequential HAT events per molecule, accounting for its superior potency relative to dihydroxylated flavonols [30]. The Nrf2/HO-1 pathway recently demonstrated for verbascoside in murine C2C12 skeletal muscle cells, including Nrf2 nuclear translocation, HO-1 upregulation, and improved mitochondrial spare respiratory capacity [36], represents a mechanistically high-priority dimension of redox signaling not yet directly investigated in any *B. globosa* experimental system. Given the high verbascoside concentrations of 63.1–112.4 mg/g DW across the included studies. Nrf2/HO-1 signaling represents a biologically relevant mechanism that warrants direct evaluation in future *B. globosa* experimental systems.

### 4.2. Anti-Inflammatory Activity: Multi-Pathway Polypharmacology

Anti-inflammatory activity in this review derives from structurally diverse compound classes: phenylethanoid glycosides, flavonoid glycosides, pentacyclic triterpenes, and sesquiterpenes. This converges on the arachidonic acid cascade through complementary mechanisms. These findings suggest a polypharmacological anti-inflammatory profile involving complementary mechanisms across multiple phytochemical classes [20,21]. The dual COX/5-LOX inhibition reported by Liao et al. [13] is pharmacologically significant because simultaneous enzymatic blockade prevents compensatory biosynthesis of alternative inflammatory mediators; a recognized limitation of selective COX-2 inhibitors in current clinical use [20,34].

The α/β-amyrin fraction reinforces the lipophilic anti-inflammatory contribution by suppressing NF-κB and inhibiting phospholipase A_2_, upstream of both the COX and LOX branches [14,20]. Torres et al. [11] provided direct cellular evidence of PGE_2_ suppression in LPS-stimulated RAW 264.7 macrophages, consistent with the NF-κB inhibitory activity of acteoside documented in independent experimental systems [31,32]; however, NF-κB pathway measurement was not performed in that study, and mechanistic attribution must be qualified as biologically plausible but not directly confirmed within the included literature. In contrast, the Becerra et al. [33] review of *P. quinquefolia* attributed anti-inflammatory mechanisms directly to modulation of the NF-κB, AP-1, and MAPK pathways. A degree of mechanistic resolution not yet achieved for *B. globosa* and representing a clear priority for future experimental work with LPS-stimulated macrophage or neutrophil models.

The anti-inflammatory profile of *B. globosa* is phylogenetically consistent with other Buddlejaceae members characterized by phenylethanoid glycosides and flavonoid complements [34,37], creating a regulatory context for clinical development that has not been documented for other *Buddleja* species in the Chilean flora, to the best of the authors’ knowledge.

### 4.3. Additional Biological Activities of Clinical Relevance

Beyond the antioxidant–anti-inflammatory axis, this review documents additional activities with clear clinical relevance. Echinacoside and linarin display hepatoprotective activity at micromolar concentrations, with linarin’s antihepatotoxic effect constituting a first report in the literature [18,37]. Verbascoside’s antiplatelet activity via inhibition of PLC-γ2/PKC-β2 is directly relevant to the cardiovascular disease burden in Chile and across Latin America [35]. Wound-healing mechanistic evidence from the induction of keratinocyte differentiation and fibroblast proteomics [16,23] provides direct molecular grounding for the dominant topical use of matico in Chilean folk medicine as it has been recently reported by Cortés [8], who highlighted the Diaguita uses of matico due its wound-healing and anti-inflammatory effects, and with forms of use such as infusions, rubs, and poultices (preferably for topical use) [8]. The selective antifungal activity against *C. albicans* [27] illustrates the bioactivity-guided nanomaterial development paradigm applicable in domestic pharmaceutical contexts, and the four novel phenylethanoid glycosides identified in that study define new pharmacological targets requiring priority characterization.

### 4.4. Translational Gap: Pharmacokinetic Barriers and Clinical Development Roadmap

The most critical translational barrier is the unfavorable oral pharmacokinetic profile of the principal bioactives. Verbascoside shows absolute oral bioavailability of approximately 4% in dogs [28,32], while echinacoside reaches only 0.83% in rats [28]. In vitro Caco-2 studies document an intestinal absorption efficiency of approximately 0.1% for verbascoside at 10–100 µM [38], reflecting overlapping barriers: incomplete paracellular absorption and sodium–glucose cotransporter (SGLT1)-mediated absorption, extensive colonic microbial hydrolysis, rapid phase II hepatic conjugation, and rapid systemic elimination [28,32,39].

Three complementary strategies are proposed: (i) Nanoformulation—chitosan-coated acteoside liposomes achieved 217.62% relative bioavailability versus free acteoside in rats [28]; co-delivery in liposomal form has shown synergistic preclinical efficacy [40]; and the PCL electrospun nanofiber platform used by Hermosilla et al. [27] confirms Chilean operational capacity for phenylethanoid glycoside nanoformulation. (ii) Colonic metabolite pharmacology—microbial biotransformation of verbascoside produces hydroxytyrosol, caffeic acid, and homovanillic acid [41,42], which have independent antioxidant and anti-inflammatory activities with substantially higher systemic bioavailability; Phase I metabolomics-based pharmacokinetic studies profiling both parent compound and metabolite kinetics simultaneously are required to reinterpret the clinical relevance of the preclinical data reviewed here. (iii) Topical and mucosal delivery—this route bypasses oral absorption entirely, is directly supported by the wound-healing, antifungal, and antiplatelet evidence reviewed [10,16,23,27,43,44,45,46,47,48,49,50].

### 4.5. Limitations of This Systematic Review

This review presents four principal limitations: (1) The primary search volume originated from Google Scholar (n = 1604 of 1660 records), which indexes a broader and less standardized corpus than MEDLINE and may retrieve records with lower bibliographic precision. (2) The temporal range (1989–2026) creates analytical asymmetries between early studies (TLC, UV/IR, NMR) and recent platforms (UHPLC-MS/MS).

Several limitations of the present review should be acknowledged. First, although the available evidence supports the antioxidant, anti-inflammatory, and mucosal repair properties of *B. globosa*, there is still limited information regarding the pharmacokinetic profile (absorption, distribution, metabolism, and excretion; ADME) of its bioactive constituents in vivo. This represents an important knowledge gap, as many plant-derived polyphenols undergo extensive biotransformation in the gastrointestinal tract and liver, which may substantially alter their bioavailability and biological activity. Consequently, it remains unclear whether the reported therapeutic effects are primarily mediated by the parent compounds present in *B. globosa* extracts or by metabolites generated during intestinal and hepatic metabolism.

Second, the interaction between *B. globosa* phytochemicals and the gut microbiota has not been sufficiently investigated. Given the proposed role of *B. globosa* in intestinal mucosal protection and repair, microbiota-mediated biotransformation may be a critical determinant of its efficacy. Emerging evidence suggests that microbial enzymes can modulate the bioactivity, toxicity, and systemic effects of dietary phytochemicals. In this regard, recent work by Guo et al. highlights the relevance of targeting gut microbiota-derived β-glucuronidase to reduce enterotoxicity and systemic inflammation, emphasizing the importance of host–microbiota–phytochemical interactions in determining biological outcomes. Future studies integrating metabolomic, pharmacokinetic, and microbiome-based approaches are therefore needed to clarify the mechanisms underlying the therapeutic effects of *B. globosa* and to identify the metabolites and microbial pathways responsible for its biological activity.

A final limitation of the current body of evidence is its predominant reliance on preclinical animal models, with insufficient critical evaluation of potential biases within the available clinical data. Furthermore, the rationale for interspecies dose conversion is not adequately substantiated, which significantly limits the reliability of translational interpretations. In addition, the absence of standardized extraction protocols represents a critical methodological weakness. In particular, solvent-dependent variability (e.g., aqueous versus ethanolic extractions) can lead to substantial differences in phytochemical composition and biological activity, thereby compromising comparability across studies and weakening the overall robustness of the conclusions.

## 5. Conclusions

This systematic review provides the first comprehensive synthesis of pharmacological and phytochemical evidence regarding *Buddleja globosa*, covering 14 studies conducted over nearly 40 years. The species contains at least 27 bioactive compounds, with antioxidant and anti-inflammatory properties standing out as its most significant pharmacological activities. Current evidence supports the traditional use of *B. globosa* through experimentally validated antioxidant, anti-inflammatory, wound-healing, and antimicrobial activities. Additional therapeutic potential has been identified in areas such as hepatoprotection, antiplatelet activity, tissue repair, and antifungal applications. Furthermore, recent phytochemical studies have expanded the species’ known chemical profile by identifying previously undescribed compounds. Future research should integrate molecular pharmacology with ethnobotanical and intercultural health perspectives, particularly in communities where traditional medicinal knowledge remains active. Preserving and incorporating this knowledge into educational, biomedical, and public health settings could foster initiatives for culturally appropriate healthcare, phytopharmacovigilance, and integrative medicine. Likewise, further study is needed on mechanisms related to redox signaling, immune regulation, microbiota-mediated metabolism, and long-term safety to better evaluate the therapeutic potential of this Andean medicinal plant.

## Figures and Tables

**Table 1:** Studies  on *B. globosa* Hope (matico): plant part, extraction method, analytical technique, identified compounds, concentrations, and assay conditions (14 studies).

Study	Plant Part Used	Extraction Method	Analytical Technique	Identified Compounds	Concentrations	Assay Conditions/ Biological Assays
Houghton, P.J.; Hikino, H. [18]	Leaves	Aqueous extract: 50 g fresh leaves boiled 10 min; filtered; freeze-dried (yield: 3.49 g). Charcoal adsorption/gradient elution (H_2_O; H_2_O:MeOH 1:1; hot MeOH; 5% NaOH). Compound isolation by DCCC (CHCl_3_:MeOH:H_2_O 43:37:20) and preparative centrifugal TLC (chromatotron). Purity confirmed by crystallization and TLC.	TLC (silica gel GF_254_) UV spectrophotometry IR spectrophotometry (Perkin-Elmer 298; Nujol) ^1^H-NMR (Bruker WM 250 MHz; D_2_O/CDCl_3_; 2D-COSY) HRMS (AEI MS 902; 35–70 eV; direct inlet)	Acteoside (verbascoside; PP1; 186 mg) Echinacoside (PP2; 125 mg) Linarin (F1; 31 mg) Catalposide (IR3; 82 mg) Catalpol (IR4; 57 mg) Aucubin (IR5; 41 mg) 6-p-Methoxycinnamoylaucubin (IR1; 18 mg; novel) 6-p-Methoxycinnamoylcatalpol (IR2; 33 mg; novel)	Compounds tested at 0.01, 0.1, and 1.0 mg/mL. Glycyrrhizin at 1.0 mg/mL as positive control.	Antihepatotoxic activity in vitro: cultured rat hepatocytes exposed to CCl_4_, galactosamine (GalN), or complement-mediated cytotoxic system (CMC).
Liao, Y.; Houghton, P.J.; Hoult, J.R.S. [13]	Roots	CHCl_3_ extract; isolation by Sephadex LH-20 column chromatography.	CC NMR (^1^H, ^13^C) HREIMS HMBC COSY NOESY	Buddledines A, B, C Dihydrobuddledine A Buddledonas A, B Zerumbone	% Inhibition COX at 50 µg/mL: 86.5 ± 5.5 % Inhibition 5-LOX at 50 µg/mL: 100	Anti-inflammatory activity: [^14^C]-arachidonic acid metabolism in rat peritoneal leukocytes. Enzyme inhibition showed IC_50_ values of 12.0 ± 0.5 µM (COX-1) and 0.8 ± 0.1 µM (COX-2), demonstrating a 15-fold higher selectivity for COX-2.
Mensah, A.Y. et al. [16]	Leaves	Aqueous extract from fresh leaves boiled in water; charcoal fractionation; Sephadex LH-20 column chromatography.	TLC (silica gel; UV 254/365 nm) UV-Vi’s spectrophotometry Sephadex LH-20 CC MS	Verbascoside (acteoside) Echinacoside Linarin Luteolin 6-Hydroxyluteolin	Individual compound concentrations not reported; extract tested at 0.001–1.0 mg/mL.	Fibroblast proliferation (Neutral Red assay; human dermal fibroblasts). H_2_O_2_-induced oxidative protection (Neutral Red/SRB viability assay). Malondialdehyde assay (thiobarbituric acid). DPPH radical scavenging.
Houghton, P.J. et al. [23]	Leaves	Aqueous extract from fresh leaves (boiling water); voucher specimen Bg 004, Dept. of Pharmacy, King’s College London.	TLC (silica gel) UV-Vis spectrophotometry (DPPH; 515 nm) Neutral Red assay (fibroblast viability) 2D SDS-PAGE (proteomics; ^35^S-Met labeling) BrdU incorporation (keratinocyte proliferation) Dot-blot (involucrin; K1/K10)	Not individually isolated in this study; flavonoids and phenylethanoids identified as active compound classes.	Extract tested at 1–100 µg/mL (collagen lattice); 10 µg/mL (keratinocyte assay). IC_50_ not reported.	Keratinocyte differentiation (involucrin, K1/K10 by dot-blot; NHK and HaCaT cells). Fibroblast protein expression (2D-PAGE proteomics). Collagen lattice contraction (human dermal fibroblasts).
Backhouse, N. et al. [14]	Leaves	Sequential extraction with n-hexane, DCM, and methanol.	GC-MS HPLC UV-Vis spectrophotometry	α-Amyrin β-Amyrin β-Sitosterol Luteolin Verbascoside Echinacoside	Fractions tested at 100–400 mg/kg (in vivo). Individual compound concentrations not reported. DPPH IC_50_: 8.4 µg/mL (total MeOH extract); 6.0 µg/mL (sequential MeOH fraction); quercetin reference: 1.07 µg/mL.	Analgesic activity (acetic acid writhing test; hot plate test; mice). Anti-inflammatory activity (carrageenan-induced paw edema; guinea pigs; 600 mg/kg p.o.; 61.4% inhibition). Topical ear edema (α/β-amyrins 3 mg/20 µL; TPA-induced 79.0%; AA-induced 47.7%; mice). Antioxidant: DPPH IC_50_; TBARS/MDA; xanthine oxidase inhibition.
Backhouse, N. et al. [15]	Leaves	Methanolic extract; liquid–liquid partition (hexane, EtOAc, n-BuOH, H_2_O).	HPLC-DAD UV-Vis spectrophotometry LC-MS	Verbascoside (acteoside) Forsitoside B Luteolin 7-O-glucoside Luteolin Apigenin	Verbascoside: 87.1 mg/g DW Forsitoside B: 55.4 mg/g DW	The Enzyme inhibition showed IC_50_ values of 12.0 ± 0.5 µM (COX-1) and 0.8 ± 0.1 µM (COX-2), demonstrating a 15-fold higher selectivity for COX-2. Antinociceptive activity (writhing test; mice). Pharmacological antagonism: naloxone (opioidergic), cyproheptadine (serotonergic 5-HT_3_), prazosin (α-1 adrenoceptor); writhing test (mice); verbascoside 67.6% vs. ibuprofen 50.0% at equimolar dose.
Fuentes, R.G. et al. [10]	Leaves	Methanolic extract (dried, powdered leaves); SPE-C18 enrichment.	HPLC-DAD-ESI/MS UHPLC-MS/MS Folin–Ciocalteu (UV-Vis)	Echinacoside Linarin Verbascoside Forsitoside B Luteolin 7-O-glucoside Luteolin Apigenin 7-O-glucoside	Echinacoside: 75 ± 3.2 mg/g DW Linarin: 58 ± 2.1 mg/g DW Verbascoside: 87 ± 4.0 mg/g DW	Antiplatelet activity (platelet aggregation; collagen, ADP, arachidonic acid stimuli; human PRP; Born turbidimetric method). PLC-γ2 and PKC-β2 inhibition (Western blot). Calcium mobilization (β-escin-permeabilized platelets).
Suwalsky, M. et al. [24]	Leaves	Lyophilized aqueous extract.	SAXS/WAXS DSC DPPH assay Hemolysis assay (human erythrocytes)	Flavonoids and phenylethanoid glycosides (extract level; no compound isolation).	Extract tested at 0.01–1.0 mg/mL.	Antioxidant activity (DPPH IC_50_). Antihaemolytic activity (H_2_O_2_-induced haemolysis; human erythrocytes). Membrane perturbation (DPPC/DMPC liposomes; SAXS/WAXS).
Gastaldi, G. et al. [25]	Leaves	Aqueous infusion (5 g/100 mL; 90 °C; 5 min); lyophilized.	HPLC-DAD LC-ESI-MS/MS Folin–Ciocalteu DPPH ORAC	Verbascoside: 63.1 mg/g DW Forsitoside B Luteolin 7-O-glucoside Luteolin Apigenin 7-O-glucoside Acteoside isomers	Total polyphenols: 147.2 ± 4.5 mg GAE/g DW. Verbascoside: 63.1 mg/g DW.	Antioxidant activity: DPPH IC_50_; ORAC. Stability under simulated gastrointestinal digestion.
Zamorano-Aguilar, N. et al. [26]	Flowers	Aqueous and hydroalcoholic (EtOH:H_2_O 50:50 *v*/*v*) extracts; lyophilized.	UHPLC-DAD-ESI/MS Folin-Ciocalteu DPPH ORAC PLS regression (VIP analysis)	Catechin: 682.4 ± 21.3 mg/100 g DW Quercitrin (quercetin 3-O-rhamnoside) Myricetin Pinocembrin Verbascoside	Catechin: 682.4 ± 21.3 mg/100 g DW Quercitrin: 58.3 mg/100 g DW ORAC (134,147 µmol TE/100 g DW): total flower extract value; NOT attributable to isolated catechin alone.	Antioxidant activity: DPPH; ORAC. Cytoprotection in CHO-K1 cells (H_2_O_2_-induced oxidative stress; MTT assay).
Torres, R. et al. [12]	Leaves	Sequential extraction (hexane, EtOAc, MeOH); CPC fractionation.	HPLC-DAD LC-ESI-MS/MS NMR (^1^H, ^13^C; HSQC; HMBC) DPPH	Verbascoside: 112.4 ± 5.1 mg/g DW Forsitoside B Luteolin 7-O-glucoside Luteolin Diosmetin Apigenin glycosides	Verbascoside: 112.4 ± 5.1 mg/g DW (MeOH fraction).	Antioxidant activity (DPPH IC_50_). Anti-inflammatory activity (PGE2 inhibition; LPS-stimulated RAW 264.7 macrophages; ELISA).
Hernández, J. et al. [12]	Leaves	Aqueous and EtOH:H_2_O (70:30 *v*/*v*) extracts; ultrasound-assisted (30 min).	UHPLC-DAD-ESI/MS Folin-Ciocalteu DPPH ABTS Broth microdilution (MIC)	Verbascoside: 98.7 mg/g DW Forsitoside B Luteolin 7-O-glucoside Luteolin Apigenin 7-O-glucoside Echinacoside	Total polyphenols: 183.4 ± 7.2 mg GAE/g DW (EtOH:H_2_O 70:30). Verbascoside: 98.7 mg/g DW.	Antioxidant activity: DPPH IC_50_; ABTS. Antibacterial activity (*S. aureus*, *E. coli*, *P. aeruginosa*; MIC by broth microdilution).
Ceriani, F. et al. [17]	Leaves	Standardized hydroalcoholic extract BG-126. Incorporated into chitosan/hyaluronic acid/gelatin scaffolds by lyophilization.	Raman spectroscopy. SEM + Trainable Weka Segmentation (pore diameter) Resazurin assay (fibroblast viability) Drop plate method (CFU/mL; log reduction) Crystal violet (biofilm adhesion) FDA/resazurin (pre-formed biofilm viability) Response Surface	No individual compound isolation/quantification BG-126 = standardized whole extract. Raman spectral markers: quercetin, catechin, thymol, carvacrol (1670 cm^−1^).	BG-126 at formulation-specific concentration per BBD design.	Antibacterial (planktonic; log CFU/mL reduction): *S. aureus* *P. aeruginosa* Antibiofilm (crystal violet + FDA/resazurin): *S. aureus + P. aeruginosa.* Cytocompatibility
Hermosilla, J. et al. [27]	Leaves	Aqueous (M1/M3) and hydroalcoholic (EtOH:H_2_O 50:50; M2/M4) extracts; maceration 24 h + ultrasound 30 min; lyophilized. Extraction yield ≈20% (all four extracts).	HPLC-DAD-ESI/MS (YL 9100; UV 270/330 nm) ATR-FTIR (JASCO FTIR-4600; 4500–600 cm^−1^) Folin–Ciocalteu (740 nm) DPPH (Trolox equivalent; 517 nm) MTT assay (3T3-L1 fibroblasts) SEM (fiber morphology; Ym index) TGA/DSC	Forsitoside B Verbascoside Luteolin 7-O-glucoside Betonioside E (novel; C_35_H_46_O_20_) β-OH-forsitoside B methyl ether (novel; C_35_H_46_O_20_) Samioside (novel; C_34_H_44_O_19_) Stachyoside A (novel; C_34_H_44_O_19_)	Extracts M1/M2 (winter) showed higher polyphenol biomarker levels than M3/M4 (spring). PCL fibers tested at 0.1, 0.5, and 1.0 mg/mL extract. Individual compound concentrations not reported.	Antifungal (PCL nanofiber scaffold; AATCC TM100): *C. albicans*: P4 (PCL 14% *w*/*v* + 1 mg/mL extract) MIC_50_/MFC_50_ determined by broth microdilution. Antibacterial (broth microdilution): *S. aureus + P. aeruginosa*—tested, no activity observed. Cytocompatibility

Abbreviations: ABTS, 2,2′-azino-bis(3-ethylbenzothiazoline-6-sulfonic acid); ATR-FTIR, attenuated total reflectance Fourier transform infrared spectroscopy; CC, column chromatography; CMC, complement-mediated cytotoxic system; COSY, correlation spectroscopy; CPC, centrifugal partition chromatography; DCCC, droplet counter-current chromatography; DSC, differential scanning calorimetry; DW, dry weight; GAE, gallic acid equivalents; GalN, galactosamine; GPT, glutamic pyruvic transaminase; HMBC, heteronuclear multiple bond correlation; HPLC, high-performance liquid chromatography; HREIMS, high-resolution electron ionization mass spectrometry; HRMS, high-resolution mass spectrometry; HSQC, heteronuclear single quantum coherence; LOX, lipoxygenase; MIC, minimum inhibitory concentration; MS, mass spectrometry; NMR, nuclear magnetic resonance; NOESY, nuclear Overhauser effect spectroscopy; ORAC, oxygen radical absorbance capacity; PCL, polycaprolactone; PLS, partial least squares; SAXS/WAXS, small/wide angle X-ray scattering; SEM, scanning electron microscopy; SPE, solid-phase extraction; TGA, thermogravimetric analysis; TLC, thin layer chromatography; VIP, variable importance in projection.

**Table 2:** Bioactive compounds of *B. globosa* Hope (matico): chemical class, biological activities, and molecular mechanisms.

Chemical Class	Compound	Structure 2D	Biological Activity	Molecular Mechanism	Ref.
Flavone	Luteolin	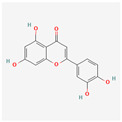	Anti-inflammatory Analgesic Antioxidant Antiplatelet	Proposed to suppress inducible COX-2 and iNOS, thereby reducing PGE2 and NO production. Inhibits PI3Kα and the thromboxane A2 receptor (TxA2-R), impairing calcium release and ERK1/2 phosphorylation in collagen-activated platelets. Antioxidant activity is attributed to ROS scavenging via the catechol B-ring (HAT mechanism). In vivo antinociceptive activity associated with inhibition of the arachidonic acid cascade (COX/5-LOX pathways).	[11,12,15,25]
	Luteolin 7-O-glucoside	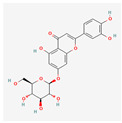	Anti-inflammatory Analgesic Antioxidant	Reported to inhibit COX-2 and iNOS, reducing PGE2 and NO in RAW 264.7 cells. Suppresses xanthine/xanthine oxidase-derived superoxide and LPS-induced hydroxyl radical generation. Antinociceptive activity has been associated with modulation of the COX/5-LOX cascade and partial modulation of µ, δ, and κ opioid receptors.	[11,12,15,23,25]
	Apigenin 7-O-glucoside	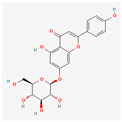	Anti-inflammatory Antioxidant	Anti-inflammatory and antioxidant activities inferred from the pharmacology of apigenin glycosides, which inhibit NF-κB-mediated pro-inflammatory cytokine expression and scavenge ROS via the apigenin aglycone. Direct mechanistic data from an isolated compound in *B. globosa* are not available.	[11,23]
	Linarin (acacetin 7-O-[α-L-rhamnopyranosyl- (1→6)]-β-D-glucopyranoside)	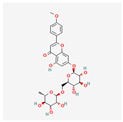	Anti-inflammatory Antioxidant Antihepatotoxic Antiplatelet	Anti-inflammatory and antioxidant activities are attributed to the acacetin aglycone (COX inhibition; reduction in prostaglandin synthesis). Antihepatotoxic in vitro: GPT reduction at 1.0 µg/mL ~41% vs. GalN and ~74% vs. CMC (comparable to glycyrrhizin; first report of antihepatotoxic activity for this compound). Glycosylation at C-7 modifies bioavailability, membrane permeability, and antiplatelet aggregation.	[10,11,13,18]
Flavonol	Quercetin	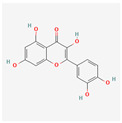	Antioxidant Anti-inflammatory	Donates hydrogen atoms to peroxyl radicals via the 3-OH and catechol B-ring (HAT mechanism), interrupting lipid peroxidation chain reactions. Chelates Fe^2+^ and Cu^2+^, preventing Fenton-type hydroxyl radical generation. Inhibits xanthine oxidase, reducing superoxide anion production. Embeds into the polar head region of membrane phospholipids, protecting lipid bilayers.	[20,23,25]
	Quercetin 3-O-glucoside (isoquercitrin)	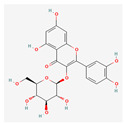	Antioxidant	Catechol B-ring of the quercetin aglycone scavenges ROS through HAT and SET mechanisms. The 3-O-glucoside moiety increases water solubility relative to the aglycone, improving bioavailability in aqueous environments.	[10]
	Quercitrin (quercetin 3-O-rhamnoside)	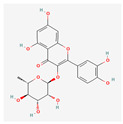	Antioxidant	Catechol B-ring donates hydrogen atoms to free radicals (HAT mechanism). VIP > 1 in PLS regression analysis identifies it as a primary antioxidant predictor in *B. globosa* flower extracts.	[26]
	Myricetin	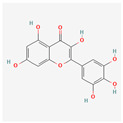	Antioxidant	The 3′,4′,5′-trihydroxy B-ring confers the highest radical-scavenging capacity among flavonols detected in *B. globosa*, enabling multiple HAT events per molecule. VIP > 1 in PLS model confirms it as a significant antioxidant contributor in flower extracts. Reduces intracellular ROS in cell-based assays.	[26]
Flavan-3-ol	Catechin	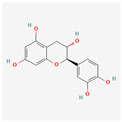	Antioxidant Chemoprotective	The catechol B-ring interrupts radical chain reactions by H-atom donation (HAT mechanism). Chelates Fe^2+^, preventing the Fenton reaction and hydroxyl radical formation. Cytoprotective in CHO-K1 cells against H_2_O_2_-induced oxidative damage.	[17,26]
	Epicatechin	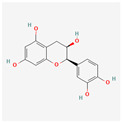	Antioxidant	Catechol B-ring scavenges ROS via HAT; chelates Fe^2+^, reducing Fenton-derived hydroxyl radicals. Higher hydrophobicity relative to catechin favors deeper membrane incorporation, protecting erythrocyte and fibroblast membranes. Cytoprotective in CHO-K1 cells.	[26]
Flavanone	Pinocembrin	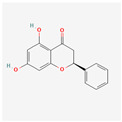	Antioxidant Antibacterial	5,7-Dihydroxy substitution pattern enables electron donation to free radicals. Antibacterial effect attributed to disruption of bacterial membrane integrity, altering permeability and inducing cell lysis.	[26]
Phenylethanoid glycoside	Verbascoside (acteoside)	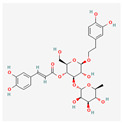	Anti-inflammatory Analgesic Antioxidant Antiplatelet	Acts as an efficient electron and hydrogen donor, scavenging ROS and reducing oxidative membrane damage. Inhibits NO release in LPS-stimulated cells (iNOS suppression). Inhibits histamine- and bradykinin-induced contractions (anti-inflammatory in vivo). Antinociceptive effect exceeding ibuprofen in the writhing test, via COX/5-LOX cascade and partial opioid receptor modulation. Antiplatelet activity via interference with TxA2 synthesis and competitive PKC inhibition.	[11,12,15,25,27]
	Forsitoside B	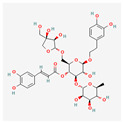	Anti-inflammatory Antioxidant Antimicrobial	One of three major compounds identified by HPLC in *B. globosa* leaf extracts across multiple seasonal collections. Anti-inflammatory activity is proposed to involve modulation of COX-mediated prostaglandin synthesis inhibition and NF-κB pathway suppression; antioxidant activity via the caffeoyl moiety (ortho-dihydroxyl catechol group). Antimicrobial properties attributed to its phenylpropanoid scaffold.	[5,8,10,11,14]
	Echinacoside	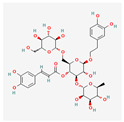	Antioxidant Antihepatotoxic Antiplatelet	Caffeoyl residue (ortho-dihydroxyl catechol group) is considered the principal antioxidant pharmacophore, contributing to ROS scavenging. Antihepatotoxic in vitro: identified as a major hepatoprotective contributor alongside acteoside given its higher abundance relative to linarin. Isolated yield: 125 mg; quantified at 75 ± 3.2 mg/g DW in antiplatelet-active leaf extract. Antiplatelet: PLC-γ2/PKC-β2 pathway.	[11,12,15,25,27]
	Betonioside E †		Antioxidant	Newly identified in *B. globosa* (C_35_H_46_O_20_). Antioxidant properties attributed to phenolic hydroxyl groups enabling effective radical neutralization. Related species reports support cytoprotective potential. Direct mechanistic data from an isolated compound in *B. globosa* not yet available.	[27]
	β-OH-forsitoside B methyl ether †		Anti-inflammatory Antimicrobial	Methylated forsytoside B derivative with additional hydroxyl group, newly identified in *B. globosa* (C_35_H_46_O_20_). Methylation may improve chemical stability and modify bioavailability. Forsytoside derivatives described as effective anti-inflammatory and antimicrobial agents in related species. Direct mechanistic data in *B. globosa* not yet available.	[27]
	Samioside †	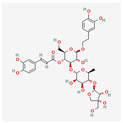	Antioxidant Cytoprotective	Structural isomer of forsitoside B, newly identified in *B. globosa* (C_34_H_44_O_19_). Shares phenylpropanoid base of forsitoside B; distinct conformation may confer differences in biological activity. Cytoprotective and antioxidant effects suggested from related species; specific mechanism requires further characterization.	[27]
	Stachyoside A †	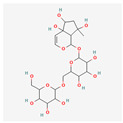	Antioxidant	Isomer of forsitoside B, newly identified in *B. globosa* (C_34_H_44_O_19_). Antioxidant activity and capacity to protect against free radical-induced oxidative damage reported in *Stachys sieboldii*. Presence complements the bioactive phenylpropanoid spectrum of *B. globosa.*	[27]
Iridoid glycoside	Aucubin	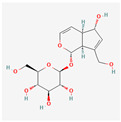	Antihepatotoxic (in vivo only)	Iridoid glycoside isolated from *B. globosa* leaves. Significant antihepatotoxic effect in whole-animal studies; not active in the in vitro cultured hepatocyte system, suggesting activity requires in vivo biotransformation. No contribution to antioxidant or anti-inflammatory effects in *B. globosa* extracts reported.	[18]
	Catalpol	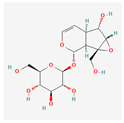	Weak antihepatotoxic (in vitro)	Isolated from *B. globosa* leaves. Weak but statistically significant GPT reduction at 1.0 µg/mL vs. CCl_4_. No significant activity vs. GalN.	[18]
	6-p-Methoxycinnamoylaucubin (novel compound)		Antihepatotoxic (in vitro)	Novel iridoid esterified with p-methoxycinnamic acid at C-6 (C_25_H_35_O_11_). Significant GPT reduction at 1.0 µg/mL vs. GalN. The p-methoxycinnamoyl substituent likely contributes to hepatoprotective activity. First reported from *Buddleja*.	[18]
	6-p-Methoxycinnamoylcatalpol (novel compound)		Antihepatotoxic (in vitro)	Novel 7,8-epoxy iridoid esterified with p-methoxycinnamic acid at C-6 (C_25_H_35_O_12_). Weak but significant GPT reduction at 1.0 µg/mL vs. CCl_4_. No significant activity vs. GalN. First reported from *Buddleja*.	[18]
Triterpenoid	α-Amyrin/β-Amyrin	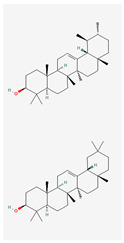	Anti-inflammatory Analgesic	Pentacyclic triterpenes isolated from the hexane/DCM fraction of *B. globosa* leaves. Analgesic activity in vivo (acetic acid writhing test; hot plate; mice). α/β-Amyrin mixture is synergistically more active than individual compounds. α/β-amyrins act through phospholipase A_2_ inhibition, with NF-κB/MAPK pathway suppression proposed as the upstream mechanism based on evidence from related triterpene systems; this pathway was not directly measured in *B. globosa*.	[14]
Phytosterol	β-Sitosterol	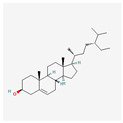	Anti-inflammatory	Phytosterol present in the lipophilic fraction of *B. globosa* leaves. Anti-inflammatory effects are proposed to involve modulation of arachidonic acid metabolism (COX pathway) and immunomodulation. Structural similarity to cholesterol enables membrane incorporation, modulating membrane fluidity and receptor-mediated signaling.	[14]
Sesquiterpenoid	Zerumbone	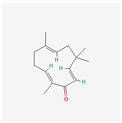	Anti-inflammatory	Monocyclic sesquiterpene isolated from *B. globosa* roots. Anti-inflammatory activity mediated by inhibition of 5-LOX (100% at 50 µg/mL) and COX pathways in rat peritoneal leukocytes. Acts as a Michael acceptor capable of modifying protein thiols, potentially suppressing IKKβ and NF-κB-dependent gene expression.	[13]
Diterpenoid	Buddledines A, B, C; Dihydrobuddledine A; Buddledonas A, B	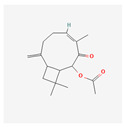	Anti-inflammatory	Novel clerodane diterpenoids isolated from *B. globosa* roots. Buddledine A (and mixture) showed combined % inhibition of COX at 50 µg/mL: 86.5 ± 5.5 and 5-LOX: 100% in rat peritoneal leukocytes. Activity attributed to inhibition of the eicosanoid synthesis cascade.	[13]

*† Compounds newly identified in B. globosa*. 2D structures were retrieved from the PubChem open chemistry database (National Library of Medicine, U.S. National Institutes of Health; https://pubchem.ncbi.nlm.nih.gov); Abbreviations: 5-LOX, 5-lipoxygenase; COX, cyclooxygenase; DW, dry weight; ERK, extracellular signal-regulated kinase; GalN, galactosamine; GPT, glutamic pyruvic transaminase; HAT, hydrogen atom transfer; iNOS, inducible nitric oxide synthase; IKKβ, inhibitor of NF-κB kinase subunit beta; LPS, lipopolysaccharide; MAPK, mitogen-activated protein kinase; MMP, matrix metalloproteinase; NF-κB, nuclear factor kappa-B; NO, nitric oxide; ORAC, oxygen radical absorbance capacity; PGE2, prostaglandin E2; PI3Kα, phosphatidylinositol 3-kinase alpha; PKC, protein kinase C; PLC-γ2, phospholipase C gamma 2; PLS, partial least squares; ROS, reactive oxygen species; SET, single electron transfer; TxA2-R, thromboxane A2 receptor; VIP, variable importance in projection.

## Data Availability

All data supporting the reported results are contained within this article and its.
